# A Review of Artificial Intelligence-Based Gait Evaluation and Rehabilitation in Parkinson’s Disease

**DOI:** 10.7759/cureus.47118

**Published:** 2023-10-16

**Authors:** Purvi L Jadhwani, Pallavi Harjpal

**Affiliations:** 1 Department of Neuro-Physiotherapy, Ravi Nair Physiotherapy College, Datta Meghe Institute of Higher Education and Research, Wardha, IND

**Keywords:** gait training, gait assessment, ai & robotics in healthcare, balance, parkinson’s disease

## Abstract

Parkinson's disease (PD) is a long-term degenerative disease of the central nervous system that affects both motor and non-motor functions. In most cases, symptoms develop gradually, with non-motor symptoms increasing in frequency as the condition progresses. Tremors, stiffness, slow movements, and difficulty walking are some of the early symptoms. There may be problems with cognition, behavior, sleep, and thinking. Dementia caused by PD becomes more common as the disease progresses. The development of PD is linked to certain sequences of motion that eventually contribute to diminished function. Patients with Parkinson's disease (PWPD) have a sluggish, scattered gait that is accompanied by intermittent freezing of gait (FOG), in which efficient heading briefly pauses. In individuals with severe PD, FOG is a neurological deficit that is related to falls and has an unfavorable impact on the patient's standard of living. Artificial intelligence (AI) and ambient intelligence (AmI) are inextricably linked as intelligence is the ability to gain new information and employ it in novel contexts. The ambience is what accompanies us, while artificial represents something developed by humans. Wearable technologies are being designed to recognize FOG and support patients in the beginning to walk again via periodic cueing. The article proposes a unique automated approach for action description that utilizes AI to carry out a non-intrusive, markerless evaluation in real-time and with full robotics. This computerized method accelerates detection and safeguards from human error. Despite significant improvements brought about by the advent of novel technologies, the available assessment platforms still fail to strike the ideal equilibrium among expenditure, diagnostic precision, velocity, and simplicity. The value of the recommended approach can be seen through a comparison of the gait parameters collected by each of the motion-tracking gadgets.

## Introduction and background

Parkinson's disease (PD), one of the oldest and most prevalent neurological disorders, exhibits a wide range of traits that could be dangerous signs and symptoms, such as tremors, bradykinesia, and rigidity in the upper body [1]. It is a serious medical condition that degrades one's quality of life for numerous causes, not the least of which is a degree of axial deterioration (gait complications, unpredictability, accidents from falls, and other injuries associated with falls). The neurological disorder dubbed Parkinson's continues to spread quickly around the entire world. As of 2020, there were currently over one million the population living with PD, imposing a $52 billion annual financial burden on the community. No supplements have been able to put an end to the course of the illness thus far. A noteworthy challenge to the advancement of PD pharmaceuticals and therapies is the absence of adequate diagnostic biomarkers. Clinical indications, primarily characterized by motor functions including tremors and rigidity, are commonly utilized to diagnose the condition [2]. No trustworthy biomarkers are existing currently for diagnosing PD or keeping track of its progression over time. There are various artificial intelligence (AI) models that are used to investigate and recognize PD such as sensory-based technology, smartphone applications, and machine learning methods, and its progression via nocturnal breathing data [2].

PD is predominantly recognized by bradykinesia, rigidity, resting tremors, alterations to posture, and gait anomalies. At the beginning of PD, cognitive and behavioral abnormalities, which include dementia, being detected throughout the disease course, and motor symptoms take place. Additional movement-related PD examinations include communication and typing. The majority of PD patients typically experience micrographia, a phenomenon where strokes are smaller than usual as a result of a deterioration in handwriting. The spiral drawing test has been frequently used in the literature for PD detection-based intelligence [1]. It could affect gait, leading to difficulties with mobility and injuries. Early detection and storage of recurrence predictors are essential to reduce issues and treat the disease [1]. The computer-assisted testing, assessment study findings, and supervision of neurological mobility conditions of the Parkinsonian type make significant use of AI, particularly machine learning [3]. Gait analysis is an efficient technique to acquire quantitative information on movement deficits in PD and plays a vital part in maintaining human mobility and health care. In the early stage, an accurate classification of PD may not be established while fundamental neuroimaging techniques do not provide differentiating signs that would allow for a PD diagnosis. Gait analysis can help in the identification of PD because discomfort and unstable posture, both significant PD signs and symptoms, impact gait patterns. Analysis of gait has been intensively used for movement problems in both individuals with PD and healthy controls [4].

Prevalence

PD affects over 10 million individuals around the world and is one of the most prevalent neurological diseases. Concerning the preceding generation, the results show an increase of 2.5 in prevalence [5]. In a study analyzing the efficacy of mainstream clinical diagnostic approaches involving post-mortem neuropathological research, both the specificity and the sensitivity of a medical diagnosis of PD were determined to be 88% and 68%, respectively [3].

## Review

Study selection

**Figure 1:**
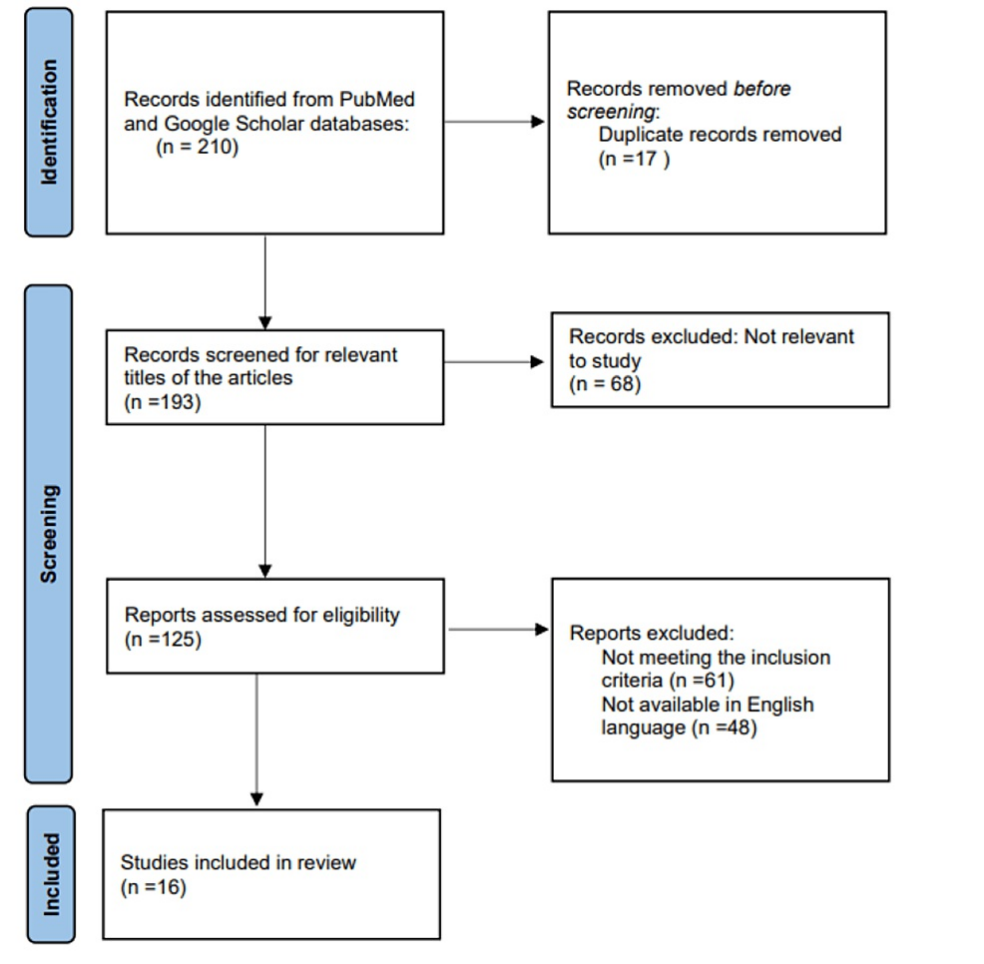
PRISMA Flow diagram PRISMA: Preferred Reporting Items for Systematic Reviews and Meta-Analyses

To assess the effects of various AI-based gait evaluations in PD, a comprehensive literature search was conducted on Google Scholar and PubMed for randomized, non-randomized clinical trials and review articles. Between Jan 2007 and Jan 2023, articles with the keywords "AI & robotics in healthcare", "gait", "balance", "assessment" and "Parkinson’s disease" were searched. A total of 210 published articles were identified. Sixteen of the 210 articles could be read in full. Studies that were included were those with patients of PD, where AI was used as an adjunct for assessment purposes, articles in the English language, and physiotherapy assessment. Exclusion criteria included any other forms of assessment and articles in other languages. Figure 1 represents the Preferred Reporting Items for Systematic Reviews and Meta-Analyses (PRISMA) flow diagram.

Implementation of AI in the assessment of clinical features

PD is frequently thought of as an instance of "movement disorder," though in addition to the typical motor signs, individuals can also struggle with an assortment of other medical conditions [5]. Remote monitoring of symptoms of PD is growing increasingly common as technological advances enable individuals to regain control of their condition at home. Patients can be analyzed within their homes, thanks to digital technological advances such as smart devices, sensors, and other devices [5]. Stretching is a way to minimize flexor muscle shortening and can assist with PD's overly flexed posture. Balance exercises may strengthen postural control (individually or in combination with additional exercise methods), minimizing the possibility of falling [6]. The primary gait abnormality referred to as "freezing of gait" (FOG), which is prominent in PD's early and late stages, becomes tougher to move and raises the risk of collapsing. By integrating gait analysis and assistive technology, devices with sensors have been utilized to recognize and predict FOG to prevent falls or minimize their effects [7]. Gait festination is a prominent condition that often adversely impacts a person's quality of life [8]. People with PD suffer from festination and FOG, which are not as common as abnormalities in gait that lead to dysfunction and collapse. In PD, basal ganglia degeneration results in deficiencies in sensorimotor coordination (hypokinesia), but altered motor cue generation causes an ordered effect, whereby movements get progressively less complex as in festination [9].

Pathophysiology

PD is a progressive neurological condition that destroys dopamine-producing neurons in the substantia nigra, a part of the brain. Dopamine is a chemical messenger that aids in controlling reward, motivation, movement, and mood. PD symptoms include tremors, rigidity, slowness of movement, and balance issues that start to show themselves when dopamine levels drop [8]. The basal ganglia regulate muscular tone, balance, coordination of opposing muscle groups, and the shift in condition required for muscles to transition from rest to motion. Additionally, some muscles can be kept at rest while others are in motion thanks to control from this area of the brain. In a healthy state, impulses travel from the brain's motor cortex through the spinal cord and reticular formation before reaching the numerous muscles that need to contract. While this is going on, other impulses go a different route through the basal ganglia, where the nerve signal is muted (toned down) to prevent jerky contractions [9]. The neurotransmitter that dampens the motor signal is dopamine, which is located in the basal ganglia. Another neurotransmitter, acetylcholine, counteracts the effects of dopamine if the dampening effect becomes excessively powerful, keeping the strength of the signals transmitted to the muscle in balance. PD impairs the proper operation of the neural pathway that regulates muscular action by causing atrophy of the basal ganglia and harm to the substantia nigra's dopamine-producing cells [5]. An excessive amount of muscle tension results in tremors and inflexible joint movement. This dysfunction also causes the body's movements to slow down. Drug therapies aim to either raise dopamine levels or prevent the release of acetylcholine, which is a dopamine antagonist [7]. One environmental element that could harm the basal ganglia is an unidentified hazardous substance. Another theory holds that the deterioration results from an early viral infection. PD may develop after encephalitis or another type of brain damage [10]. In the end, dopamine-deficient basal ganglia network neurons display unsteadiness and unaware movement. Equilibrium problem has been linked to falling, having reduced mobility, and having a lower quality of life for sufferers [10].

Assessment of gait via AI

The signal patterns gathered by the provided physiograph emphasize the variations in the stance phases with the left and right foot, as demonstrated by the kinematic gait variables. According to certain research, stance phases are shorter in patients with PD than in healthy people [8]. Additionally, they have decreased heel strike and toe-off angles, which point to a lack of dorsiflexion and plantarflexion of the ankle. These changes could be brought on by stiffness, bradykinesia, and poor balance control. The differences between the stance phases with the left and right foot could be an indication of PD's asymmetry [4]. On one side of the body more often than the other, some persons with PD experience more severe symptoms. Their risk of falling may be impacted, as well as the symmetry and stability of their gait [5].

Flat feet, which are typical of the initial and middle stages of PD, were an interesting trait of the patients. Plantar pressure, lower-limb muscle contractions, and arm balance all play a role in a functional gait pattern, and this can be determined using the correlation function. These characteristics are connected to gait stability, symmetry, and smoothness. When walking, the left and right feet's plantar pressure patterns can be compared using the correlation function, as can the muscular activity in the arms and lower limbs [2]. A strong association or similarity is indicated by a high correlation, whereas a weak relationship or dissimilarity is shown by a low correlation. Gait analysis can identify and measure the gait deficits brought on by PD, such as shorter steps, more variable steps, reduced arm swing, and greater postural sway, by applying the correlation function [9].

In recent studies, convolutional neural networks (CNN) have been utilized to provide AI support for decisions for determining the presence of PD gait patterns. CNNs are a subset of deep learning systems that are capable of processing images and extracting valuable information from them. CNN can be used in the diagnosis of PD to examine various types of pictures, such as brain scans, spiral diagrams, or samples of handwriting, and find small alterations that could point to the disease's presence. One study classified the EEG signals of PD patients and healthy subjects using CNN and in another CNN was used to identify PD in spiral drawings, which are known to be impacted by the disease's motor symptoms [9,10]. These techniques may increase the precision and effectiveness of PD diagnosis and aid in tracking the disease's progression. The accuracy, sensitivity, specificity, and precision of the CNN model are calculated as positive predicted values (PPV), which are additionally referred to as accuracy (Acc), sensitivity (Se), specificity (Sp), and precision. The percentages of true positive (TP), false positive (FP), true negative (TN), and false negative (FN) have been employed for describing these, respectively [10]. A prevalent and immobilizing sign of PD is a disrupted gait. Nevertheless, gait is not usually evaluated statistically and is rather described using broad terms that are responsive to modifications carried through the progress of a disease. The sensitivity of gait assessment has been enhanced by assessing multiple gait characteristics (such as velocity, variations, and tilt) under genuine and more demanding circumstances (such as dual tasking, turning, and everyday living) [11]. It is crucial to evaluate the dynamic as well as static balance, whether or not a cognitive activity is involved. A force platform is utilized in a digital posturography evaluation to determine the response forces the human physique generates on the ground's surface and the movement of the centre of pressure [12].

Gait training

Conventional rehabilitation is advised with the aim of retaining stability among those with PD [13]. Recently, novel information regarding turning, axial deviations sensorimotor integration disabilities, and neuroimaging have emerged. Turning difficulties are frequently noted by PD patients, and knowing the association between FOG and hip fractures, these turning difficulties are of substantial beneficial significance. It's shocking to discover that standing and walking are not entirely automatic procedures governed by subcortical neural networks, demanding little to no effort from the mind [14]. Normal walking patterns and postural stability are maintained by subcortical systems, including the basal ganglia. These structures produce subconscious internal cues that start movement sequences.

For people with mild to severe PD, retaining independent motor function and the capacity to walk is a crucial therapeutic goal. The modified strategy to achieve this goal is to help patients maintain their mobility for as long as possible, prevent them from becoming immobile too soon, and improve their quality of life. Strength training may boost the endurance of muscles in particular, and it additionally improves gait performance. Stretching can reduce flexor muscle shortening, which will ease PD's excessively flexed posture. Balance exercises may enhance postural control (alone or in conjunction with other training modalities), which lowers the chance of falling [1]. CNNs in particular have made considerable use of the pooling methodologies in their deep learning strategies. Artificial neural networks of the sort known as CNNs are used to analyze and extract features from photographs. They take their cues from the biological design and operation of the brain's visual cortex, which is made up of neurons that react to various patterns of light in the visual field. CNNs are frequently used in computer vision for a variety of applications, including image classification, segmentation, localization, and detection.

The proposed method consists of three steps: data acquisition, feature extraction, and classification. In the data acquisition step, the authors use a wearable device called GaitTrack (University of Illinois, USA) to collect gait signals from 100 subjects, including 50 PD patients and 50 healthy controls. In the feature extraction step, the authors use a combination of empirical mode decomposition (EMD) and CNN to extract the salient features from the gait signals. EMD is a signal processing technique that decomposes a signal into a set of intrinsic mode functions (IMFs) that represent different frequency components of the signal. CNN is a deep learning technique that uses multiple layers of filters to learn hierarchical features from the input data. The authors claim that EMD and CNN can capture the nonlinear and nonstationary characteristics of the gait signals. In the classification step, the authors use a long short-term memory (LSTM) network to classify the extracted features into four classes: normal, mild PD, moderate PD, and severe PD. LSTM is a type of recurrent neural network (RNN) that can handle sequential data and learn long-term dependencies. The authors claim that LSTM can model the temporal dynamics of the gait signals and improve the accuracy of classification [15]. For the assessment of the individual activation techniques and technologies for frozen gait identification, people designed a prototype continuous tracking system. The goal is to create a minimally intrusive system with a long lifespan of batteries for ideal user convenience [16].

A study by Tripoliti et al. provides a computer-assisted methodology for determining the presence of FOG events (both long-term and short-term). Data extracted from gyroscopes and accelerometers attached to individuals' limbs can be utilized to assess the procedure [17]. The potential of implementing the harmonic bands of distal upward vertical acceleration to differentiate FOG from movements or voluntary standing. High-frequency elements in the 3-7 Hz (freeze) spectrum of vertical leg motion are linked with freezing while commencing a stride, rotating, or approaching across an obstruction, matching the "trembling" observed while FOG within this present investigation and earlier research [18]. The individuals' capacity to diminish the duration of the period utilized outdoors to decrease body sway and area of no return during the steady state phase has been demonstrated to be the most significantly affected by vibrotactile feedback [19]. The difference that exists between balanced gait and Parkinsonian gait is readily discernible. Decreased mobility constitutes one of the most emblematic yet undifferentiated initial signs of Parkinsonian gait. Initial PD individuals had been observed to experience diminished arm motion magnitude, fluidity of motion, enhanced interlimb imbalances, and other characteristics that are more characteristic of PD and are often initial motor manifestations. The PD distinguishing characteristics are resting tremors, bradykinesia, and rigidity; despite this, aberrant alignment and movement might be observed in the initial phases of the illness. Another of the primary features of PD gait is believed to be a brief decrease in stride length. Evaluation of spatiotemporal gait parameters has not been demonstrated to have been efficient as a variable assessment for recognizing and detecting gait anomalies. On the contrary, the outcomes of research by Ileșan et al. suggested that the initial intermediate PD patients had decreased spatiotemporal gait characteristics (speed and cadence), that were reinforced by comprehensive sub-item analysis of various gait parameters [10]. The gait statistics were evaluated using Mobishoe i.e., deep brain stimulation (DBS) off-medication off state, DBS stimulation off-medication on, and DBS stimulation on and on medication for every one of the four scenarios. While most people were administered a drug known as levodopa while receiving implantable pulse generator (IPG) stimulation of any kind, the findings on the assessment of spatial features indicate a substantial increase in average step length and step height on each side [20]. In PD, substantially greater motor indicators and a decreased quality lives correspond to anxiousness. Considering discrepancies in the system responsible for producing dopamine and other interrelated components like the noradrenergic and serotonergic platforms participate in these pathologies, many studies support the hypothesis the fact these pathologies exhibit underlying biological mechanisms that constitute the origin of anxiety symptoms in PD [21]. The virtualization of rehabilitation is an instance of present-day innovations in technology. Diagnostics, treatment, and educational studies of patients all comprise such technology. Recognizing how to implement virtual reality (VR) and motor imagery (MI) into rehabilitation plans of care demands study, and as our understanding of VR and MI has grown, so has our ability to do research in this area [22]. The invention of symptomatic treatment that targets common denominators, primarily dopamine insufficiency has drawn advantages from dealing with PD as a single disease, but efforts to alter the course of the underlying condition have been pointless because customized biological targets may be pathogenic in a minority of individuals but not in the majority of those who are affected.

Neurorehabilitation capitalizes on motor learning, referring to one's ability to improve and retain achievement via practice. Subcortical structures, including the basal ganglia, which are among the brain areas crippled by PD, are vital for motor learning. As a consequence, it is not strange that individuals with PD often suffer from impaired motor learning [23]. Table 1 provides the matrix of the articles selected for the review.

**Table 1:** Matrix of the articles selected for review PD: Parkinson's disease, VR: virtual reality, FOG: freezing of gait, FOGC: freezing of gait criterion, FI: freeze index, VRG: virtual reality glasses, PSD: power spectral distribution, H & Y: Hoehn and Yahr Scale, UPDRS: Unified Parkinson's Disease Rating Scale

S. no	Author, Year	Journal	Type of article	Main Finding/Methods	Conclusion
1	Chung et al., 2017 [24]	IEEE Virtual Journals	Review article	Twenty-five FOG episodes are incorporated in the data, with total and average time spent running of 100 and 3.622.91 seconds, respectively. A majority of 80% of all FOG events were less than 5 seconds, and the proportion of the entire FOG period to extend of facts assembled was estimated to be 14.55%.	Based on the investigation's analysis of data that was collected, FOG has greater accuracy in recognizing FOG episodes than FI and FOGC, which ends up resulting in fewer false positive and false negative events.
2	Yi-Tsen Pan and Han U. Yoon, 2016 [25]	IEEE	Review article	In this study, 20 healthy young individuals lacking neurological or musculoskeletal anomalies (six females and 12 males; mean age: (28.6 4.5 years) took part. Three sensory methods and two forms of cognitive stimulation were administered to the individuals while they remained stationary upon a force plate for thirty-five seconds. The three scenarios for the different senses were: (1) No impairment, (2) Visual, and (3) Vision and Vestibular Impairment.	Skin stretch data has been employed to build a viable model of a multisensory reinforcement apparatus for the regaining of motor control of posture. Though several alterations may be performed to achieve an additional enhancement of balance, Studies have revealed that the perceptual improvement caused by feedback from stretching the skin at the fingertip can improve balance as established by a range of typical postural sway motions.
3	Ferster and Mazilu, 2015 [26]	European Union Digital Library	Review article	We evaluate data as Average, deviation from the mean, and Variable Coefficient over all previous-to-FOG sessions for each of the 15 participants to evaluate whether there are significant overall patterns in gait and frequency components before FOG. The statistics are derived for each synchronized window, particularly the ones that start 5 seconds before FOG, 7 seconds before FOG, and so on.	We examined the Parkinson's disease-related gait in individuals within a short period among walking and gait freezing, in contrast to intervals before other moving actions, such as rotations. The purpose of this research is to recognize certain movements. characteristics that shift just before a FOG of gloom (FOG), enabling us to predict FOG in real-time only minutes earlier and help the individual get out of the FOG of gloom.
4	Badarny S and Peretz J, 2014 [27]	Virtual Cues in Parkinson's Patients	RCT	Only 15 of the overall 25 people whose names were chosen for the study - 9 men and 6 women having an average age of 69.25 years and a mean illness duration of 4.58 years - completed it. Of these patients, 64% demonstrated over 15% gain and 30% showed over 12%increase in their initial achievement. five of the individuals confirmed that, after 7 days, they maintained their walking on psychological tiles.	This investigation indicates that PD patients' walking abilities improve progressively after undertaking virtual reality (VR) training. The conclusion coincides with a previous instance study, which concluded that a single individual gained significantly after undergoing two months of training employing indicators of vision that were put on the ground.
5	Yoram Baram, 2013 [28]	Frontiers in Neurology	Review article	The physical movement of the person's body provides an optical signal, not the reverse way out, based on investigations on the organic sensory-motor system that underpins human locomotion in conjunction with visual stimuli. Understanding the distinction between closed-loop and open-loop neurological control of locomotion relies on this seemingly basic reality.	Utilizing sensory information to address movement disorders in patients' gait has been investigated. While a few investigations indicate that open-loop tactile stimulation enhances balance and gait, other studies have raised questions about how effective routine sensory inputs are at boosting balance and gait.
6	Griffin and Greenla, 2011 [29]	Journal of Neurology	Original Communication	The impacts of original and digital signals on each other without healthcare treatments - gait issues tend to be most severe and extend the greatest potential for improvement - and on medication were analyzed in a placebo-controlled study to figure out the practical advantages of encouragement in the participants' consistent daily pharmaceutical condition.	These outcomes reveal that certain features of mid-stage PD patients discontinuing their prescribed medicines can be optimized by establishing visual-flow signals via the VRG. However, neither of the VRG triggers distinguished up as being beneficial in a substantial number of patients.
7	Zabaleta et al., 2009 [30]	Springer	Conference paper	A neurologist at the Hospital of Donostia searched for six patients, who had been diagnosed with PD. The inclusion criteria were revised to make sure that people with PD who frequently went through FOG episodes were considered. Based on the UPDRS (Unified Parkinson's Disease Rating Scale), they received scores of 5 and 1 for gait disturbance (out of a possible 6).	Each frame of time has been split into two categories - normal gait and frozen gait - using a multidimensional linear discriminant evaluation of characteristics of recovered components obtained from the PSD. Though it fluctuates extensively from subject to subject, the obtained PSD characteristics can be applied to recognize a person's genuine walking status (FOG presence).
8	Bachlin et al., 2009 [31]	IEEE	Review article	Patients with idiopathic PD who were diagnosed with previous instances of FOG and gained the ability to walk unaided within the Off phase were enrolled in the trial. In this assessment, eight PD patients (five women) with a mean age of 60.5 4.0 years with an H&Y score in ON of 3.0 0.45 engaged. PD patients frequently demonstrate substantial amounts of fluctuations in their ability to move around.	A specialized general-purpose modular research platform was implemented in the current study. Numerous technological aspects require advancement This platform is modular; hence it could be easily modified into a system that was developed just for our job. It might be simplified into one little sensor node which demonstrates the FOG detection strategy.
9	Moore and MacDou, 2007 [32]	Science Direct	Original article	The stride monitor was tested for accuracy using nine healthy individuals (four men and five females), neither of these had a history of gait difficulties. Height was around 145 and 160 cm, ranging from 35 to 59 years old. To validate the measurement accuracy, four men and five women who were recently diagnosed with idiopathic Parkinson's disease participated in the investigation.	The outcomes of this study demonstrate that assessing stride length precisely using a single shank-mounted stride monitor can be both viable and feasible for an extended period of gait evaluation in Parkinson's disease. The integrated accelerometer/gyroscope sensor exhibited more precise measurement than previous approaches implementing solitary and numerous gyroscopes.

## Conclusions

Gait and mobility studies reinforce physiotherapist processes and their use in clinical healthcare facilities strengthens the effectiveness of therapeutic properties. Both clinical trials and studies are increasingly relying on solutions based on AI. It becomes crucial to be thoughtful about how AI and healthcare providers may collaborate to manage symptoms and cure diseases as these algorithms boost. Clinicians are currently given a formidable opportunity to keep track of symptoms through cell phones' widespread popularity and the low cost of sensors. Tools evolved to record the experiences of patients in a free-living scenario can make it possible to adjust symptomatic therapy to lessen ailments burden. By Unified Parkinson's Disease Rating Scale (UPDRS) III appraisals, posture characteristics improved after levodopa and IPG stimulation. The quantitative assessment of the spatiotemporal properties was made possible through the usage of Mobishoe. Levodopa and stimulation both had added supplementary benefits that triggered improvements in the length of the stride and step height. Parkinson's patients still have the potential to learn new movement patterns, which enables them to benefit from physical exercise that tests their balance and gait in order to prevent falls, especially in the early stages of the disease.
